# A geospatial analysis of two-hour surgical access to district hospitals in South Africa

**DOI:** 10.1186/s12913-020-05637-0

**Published:** 2020-08-13

**Authors:** Kathryn M. Chu, Angela J. Dell, Harry Moultrie, Candy Day, Megan Naidoo, Stephanie van Straten, Sarah Rayne

**Affiliations:** 1 Centre for Global Surgery, Department of Global Health, Stellenbosch University, Cape Town, South Africa; 2 Department of Surgery, University of Cape Town, Cape Town, South Africa; 3 Department of Global Health, University of Stellenbosch, Francie Van Zijl Dr Tygerberg Hospital, Cape Town, 7505 South Africa; 4 School of Pathology, University of the Witwatersrand, Johannesburg, South Africa; 5 Respiratory and Meningeal Pathogens Research Unit, Medical Research Council, Cape Town, South Africa; 6 Health Systems Research Unit, Health Systems Trust, Cape Town, South Africa; 7 Department of Surgery, University of the Witwatersrand, Johannesburg, South Africa

**Keywords:** Global surgery, Surgical access, Geographic information systems, Surgical capacity, District hospital, South Africa

## Abstract

**Background:**

In a robust health care system, at least 80% of a country’s population should be able to access a district hospital that provides surgical care within 2 hours. The objective was to identify the proportion of the population living within 2 hours of a district hospital with surgical capacity in South Africa.

**Methods:**

All government hospitals in the country were identified. Surgical district hospitals were defined as district hospitals with a surgical provider, a functional operating theatre, and the provision of at least one caesarean section annually. The proportion of the population within two-hour access was estimated using service area methods.

**Results:**

Ninety-eight percent of the population had two-hour access to any government hospital in South Africa. One hundred and thirty-eight of 240 (58%) district hospitals had surgical capacity and 86% of the population had two-hour access to these facilities.

**Conclusion:**

Improving equitable surgical access is urgently needed in sub-Saharan Africa. This study demonstrated that in South Africa, just over half of district hospitals had surgical capacity but more than 80% of the population had two-hour access to these facilities. Strengthening district hospital surgical capacity is an international mandate and needed to improve access.

## Background

The Lancet Commission on Global Surgery (LCGS) reported that 5 billion people lack access to safe, timely, and affordable surgical care. In trying to define a minimum package of care for every health system, there are six indicators that are routinely measured (Table 1). The first indicator is the proportion of a population that lives within 2 hours of a facility that provides the bellwether procedures (caesarean section, laparotomy, and treatment of an open fracture), which are used by the LCGS as a proxy for surgical capacity [1]. A recent modelling of two-hour access (2HA) in sub-Saharan Africa (SSA) demonstrated large inter-country variation (23–97%). The 2HA in South Africa was estimated to be 95%, however, it did not consider the actual surgical capacity at each hospital [2].

**Table 1:** Global surgery indicators to measure universal surgical access

Indicator	2030 Targets (per country)
Two-hour access to timely surgery	Minimum 80% of the population with access to a facility that can perform a caesarean delivery, laparotomy, and treatment of open fracture (the bellwether procedures) within 2 hours
Specialist surgical workforce density	20 surgical, anesthetic, and obstetric doctors per 100,000
Surgical volume	Minimum of 5000 procedures per 100,000; 100% countries tracking
Perioperative mortality	100% countries tracking
Protection against impoverishing expenditure	100% protection against impoverishment from out-of-pocket payments for surgery and anesthesia
Protection against catastrophic expenditure	100% protection against catastrophic expenditure from out-of-pocket payments for surgery and anesthesia

Adapted from Meara JG, Leather AJ, Hagander L, Alkire BC, Alonso N, Ameh EA, et al. Global Surgery 2030: evidence and solutions for achieving health, welfare, and economic development. Lancet 2015; 386 (9993): 569–624

South Africa is an upper- middle- income country with one of the most unequal income distributions in the world [3]. Approximately 84% of the population relies on the public (government) health care system [4], which is organized around primary health care clinics (PHC) and community health centers (CHC). PHC and CHC refer patients to district, then regional and tertiary hospitals for higher levels of care [5]. The government surgical services are highly variable in terms of capacity and output and only employ 42% of general surgeons [6]. While surgical care is a component of the Department of Health Strategic Plan, implementation strategies across different hospital levels are not well outlined [7].

The World Health Organization stated that essential surgical care should be delivered at district hospitals (DH) which has been shown to be cost-effective [8–12]. However, DH surgical capacity in many SSA countries remains unmeasured.

The objective of this study was to identify the proportion of the population living within 2 hours of a district hospital with surgical capacity in South Africa.

## Methods

### Hospital selection and definitions

All government national, tertiary, regional, and district hospitals in South Africa were identified from the South African National Department of Health (NDoH). Each health district and its corresponding DH had defined district boundaries. While patients could attend other facilities in acute emergencies, we assumed that district boundaries would likely be followed for the majority of surgical referrals given defined referral pathways from PHC and CHC to a DH.

As per the LCGS’ definition of 2HA, data for all three bellwether procedures performed at DH was not readily available in South Africa. Therefore, a surgical district hospital (S-DH) was defined by the presence of a functional operating theatre, a surgical provider, and provision of at least one caesarean section (CS) annually. CS data is routinely collected by NDoH for DH facilities and was obtained for 2015–16. Data for the presence of a functional operating theatre and surgical provider was done through telephonic surveys to facility managers by one of the authors (AD) between 2015 and 2016 [13, 14].

### GPS locations

Geographical Positioning Satellite (GPS) coordinates for hospitals were obtained from the National Institute for Communicable Diseases. GPS coordinates were reviewed using logical checks and compared with NDoH datasets. Discrepancies were manually checked using a combination of Landsat images, Google Maps, Google Street View, telephone calls to facilities, and metadata from photographs.

### Population data

The 2014 population estimates for 103,576 Enumeration Areas for South Africa were obtained from the Environmental Research System Institute (ESRI, Redlands, CA) IDEAL dataset.

### Road network data

We obtained road map data for South Africa from the OpenStreetMap (OSM) project (http://www.openstreetmap.org/). Road speed limits from OSM were utilized, where available, to calculate travel time impedance. Where OSM road speed limits were not available, travel speeds of 110 km per hour (km/h) were assigned to highways, 100 km/h to regional roads, 80 km/h to regional secondary roads, 60 km/h to local roads, and 50 km/h to unclassified roads and tracks in keeping with standard OSM algorithms. Standard OSM modifications for road surface (e.g. gravel/dirt = speed/2); and road smoothness (e.g. horrible = speed/2) were incorporated. The road network was compiled in ArcMap (version 10.3) and identified errors manually corrected.

### Spatial analysis

2HA service areas for all hospitals were estimated using detailed non-overlapping polygons in the service area tool in ArcMap (version 10.3). Since the off-network travel time to the nearest road was not directly modelled, high and low 2HA estimates were generated for each analysis. Low 2HA estimates were generated by trimming the 2HA service area polygons to within 1 kilometer of the outer network edges, whereas high 2HA estimates were not trimmed resulting in larger service areas. Mask area weighting, incorporating mesozones with population counts of less than five people as mask areas, was used to estimate the proportion of the population residing within the 2HA service areas.

## Results

**Fig. 1:**
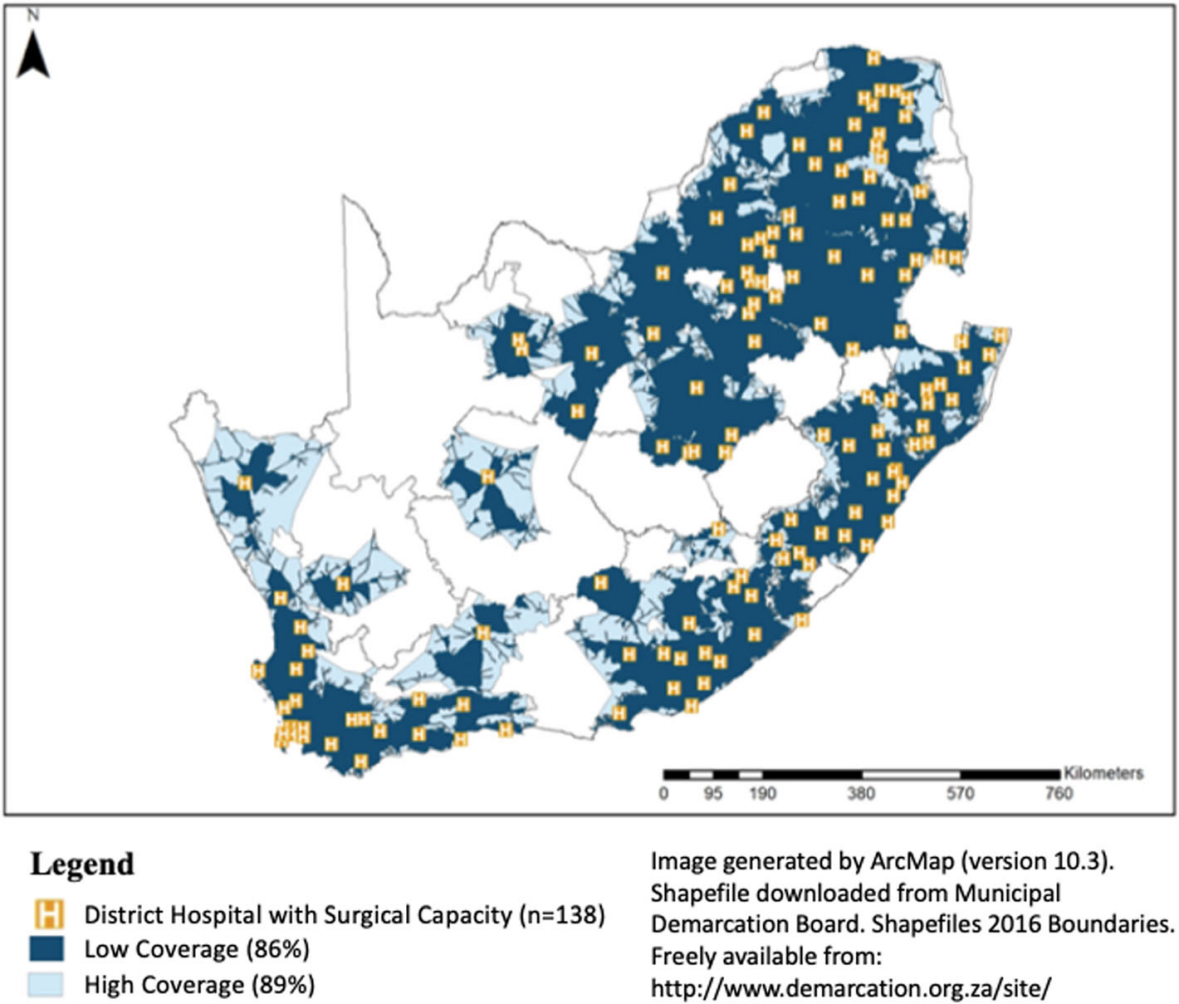
The population proportion with two-hour access to district hospitals with surgical capacity in South Africa

There were 315 government hospitals in South Africa (75 tertiary/regional and 240 DHs). Ninety-eight percent of the population lived within 2 h of one of these facilities. Although there were large areas of the country that did not have 2HA, these areas were sparsely populated covering only 2 % of the population. Of all DH, 138 (58%) could be defined as S-DH (DH with a functional operating room, a surgical provider, and performed at least one CS annually). The low and high estimates for 2HA to a S-DH were 86 and 89% respectively (Fig. 1).

## Discussion

In South Africa, an upper- middle- income country, the majority of the population lived less than 2 hours away from a district hospital with surgical capacity, exceeding the LCGS target of 80% [1]. However, almost half of district hospitals did not have surgical capacity. The World Health Assembly urged members to incorporate essential and emergency surgical care into universal health coverage, including integration “in primary health care facilities and first- referral (district) hospitals” in an unanimously passed declaration [12]. Strengthening DH surgical capacity is an international mandate, and countries are tasked with formulating national surgical plans to improve access [12]. The South African NDoH has defined a DH surgical package but this has not been widely implemented [15].

Given the lack of data for all three bellwether procedures in many low- and middle- income countries (LMIC), 2HA as defined by LCGS is challenging to measure. In a recent study, 2HA using the LCGS definition could only be calculated in 19 countries and of these, only 2 were in SSA [16]. Our specific methodology of defining surgical capacity through the provision of at least one CS annually, and the presence of a functional operating theatre and surgical provider has not been reported elsewhere but might be a practical and useful proxy in other LMIC.

Another limitation of the 2HA indicator is that it does not consider other factors that impact access, such as the availability of transport or financial constraints. For example, only 29% of households in South Africa have private cars and public transport is not reliable in every part of the country [17]. Furthermore, ambulances are not readily available and may not respond within the two-hour access window. In the rural area of Eastern Cape, there is a severe shortage of pre-hospital emergency medical services with only 12 ambulances for a population of 1 million (the recommendation is one per 10,000 persons) [18]. Financial costs can limit access to- and utilization of- health services. A recent modelling study demonstrated that combined direct medical and non-medical costs would potentially be catastrophic for up to half of the global population [19].

Our study had methodologic limitations. We did not capture the number of hours per week a surgical provider or operating theatre were available. In addition, other human resource, equipment, and implementation factors that can affect surgical capacity such as the availability of theatre/post-operative personnel and surgical materials were not measured. An in-depth situational analysis into the various barriers to strengthening surgical capacity and outputs at South Africans DH is needed. Moreover, by only including only CS and not the other two bellwether procedures, our proxy definition may have overestimated 2HA as defined by LCGS. Finally, our study did not include 2HA to private facilities. The majority of South Africans do not have private health insurance [4] but all people can access private facilities to stabilize an emergency condition prior to transfer to a public hospital [20]. The private sector contribution to 2HA for emergency surgical conditions was not measured by this study.

## Conclusion

LCGS recommended six indicators to evaluate surgical delivery, including 2HA [1]. This study demonstrated that in South Africa, more than 80% of the population could have 2HA to DH with surgical capacity. However, this indicator as a global metric may not be practical given the lack of available country-level data on bellwether procedures [16] and because it does not measure other aspects of true access. Nevertheless, surgical access is a key component of surgical equity and finding improved ways to measure and achieve it must be a global health priority.

## Data Availability

The data that support the findings of this study are available from the South African National Department of Health and National Institute for Communicable Diseases, but restrictions apply to the availability of these data, which were requested for use in the study, and so are not publicly available. Data are however available from the authors upon reasonable request and with permission of South African National Department of Health and National Institute for Communicable Diseases. The 2014 population estimates for Enumeration Areas and Road map data for South Africa are publicly available from the Environmental Research System Institute (ESRI, Redlands, CA) IDEAL dataset, and OpenStreetMap (OSM) project (http://www.openstreetmap.org/), respectively.

## Abbreviations

LCGS: The Lancet Commission on Global Surgery
2HA: Two-hour access
SSA: Sub-Saharan Africa
PHC: Primary health care clinics
CHC: Community health centers
DH: District hospitals
NDoH: National Department of Health
S-DH: Surgical district hospital
CS: Caesarean section
GPS: Geographical Positioning Satellite
OSM: OpenStreetMap
km/h: Kilometers per hour
LMIC: Low- and middle- income countries
